# HERG Protein Plays a Role in Moxifloxacin-Induced Hypoglycemia

**DOI:** 10.1155/2016/6741745

**Published:** 2015-11-16

**Authors:** Hai-Yan Qiu, Sha-Sha Yuan, Fang-Yuan Yang, Ting-Ting Shi, Jin-Kui Yang

**Affiliations:** ^1^Department of Endocrinology, Beijing Tongren Hospital, Capital Medical University, Beijing 100730, China; ^2^Beijing Key Laboratory of Diabetes Research and Care, Beijing 100730, China

## Abstract

The purpose of this study was to investigate the effect of moxifloxacin on HERG channel protein and glucose metabolism. HERG expression was investigated using immunohistochemistry. The whole-cell patch clamp method was used to examine the effect of moxifloxacin on HERG channel currents. A glucose tolerance test was used to analyze the effects of moxifloxacin on blood glucose and insulin concentrations in mice. Results show that HERG protein was expressed in human pancreatic *β*-cells. Moxifloxacin inhibited HERG time-dependent and tail currents in HEK293 cells in a concentration-dependent manner. The IC_50_ of moxifloxacin inhibition was 36.65 *μ*mol/L. Moxifloxacin (200 mg/kg) reduced blood glucose levels and increased insulin secretion in wild-type mice at 60 min after the start of the glucose tolerance test. In contrast, moxifloxacin did not significantly alter blood glucose and insulin levels in *HERG* knockout mice. Serum glucose levels increased and insulin concentrations decreased in *HERG* knockout mice when compared to wild-type mice. The moxifloxacin-induced decrease in blood glucose and increase in insulin secretion occurred via the HERG protein; thus, HERG protein plays a role in insulin secretion.

## 1. Introduction

Human ether-a-go-go-related gene (*HERG*) encodes the HERG ion channel, which is a member of the voltage-dependent potassium channel (Kv) family.* HERG* mutations reduce the outward flow of potassium during repolarization and elongate the QT interval leading to polymorphic ventricular tachycardia, cardiac syncope, and sudden death. This is known as the long-QT syndrome (LQTS) [1]. HERG ion channels are expressed in the human pancreas where it has been shown to negatively regulate insulin secretion and positively regulate glucagon secretion [2].

Fluoroquinolones are antibiotics that are effective against gram-negative bacilli, but as drug development continues, their antibacterial spectra expand. Fourth-generation fluoroquinolones, such as moxifloxacin and levofloxacin, are also effective against* Streptococcus pneumoniae*, a gram-positive cocci, and anaerobic bacteria. With the widespread clinical use of these drugs, dysglycemia has been reported as an adverse effect. gatifloxacin [3], ofloxacin [4], and lomefloxacin [5] have been reported to lead to hypoglycemia, whereas ofloxacin [4] and gatifloxacin [3] have been reported to result in hyperglycemia. Although these side effects are rare, they require hospitalization and can endanger the life of a patient; thus, patients taking fluoroquinolones should be monitored for dysglycemia.

Some fluoroquinolones cause hypoglycemia by blocking ATP-sensitive potassium channels (K_ATP_) in pancreatic *β*-cells leading to stimulation of insulin secretion [6]. By stimulating histamine secretion, gatifloxacin can indirectly induce adrenaline release and elevate blood sugar levels. Gatifloxacin can also cause vacuolization of islet *β*-cells leading to a decrease in insulin production and hyperglycemia [7]. K_ATP_ channels and other ion channels have been found to synergistically regulate insulin secretion [8]; thus, HERG potassium channels and K_ATP_ channels together may regulate insulin secretion by pancreatic *β*-cells [9]. Because the ability of moxifloxacin to inhibit HERG channels is unknown, this study explored the effect of moxifloxacin on HERG channel currents and glucose metabolism in mice.

## 2. Materials and Methods

### 2.1. Experimental Animals and Human Tissues

C57BL/6* HERG* knockout mice were created using transcription activator-like effector nucleases (TALENs). Human pancreatic tissues were obtained by surgical excision. This study was approved by the Ethics Committee of Beijing Tongren Hospital, Capital Medical University, and was performed according to the principles of the Declaration of Helsinki II. We obtained written informed consent from each participant. Animal experiments followed the national ethical guidelines implemented by our institutional Animal Care and Use Committee and were approved by the Ethical Review Committee at the Institute of Zoology, Capital Medical University, China.

### 2.2. Immunohistochemistry

Human pancreatic tissues were fixed by immersion in 4% paraformaldehyde for 2 h at 4°C and were then embedded in paraffin. Paraffin sections (5 *μ*m) were pretreated for antigen retrieval using a microwave. Paraffin sections were incubated with primary antibody, goat anti-insulin antibody (diluted 1 : 100, Santa Cruz Biotechnology Inc., CA, USA) or rabbit anti-HERG antibody (diluted 1 : 200, Santa Cruz Biotechnology Inc., CA, USA), overnight at 4°C. The sections were washed three times with phosphate-buffered saline (PBS) and then were incubated with Alexa Fluor 555 donkey anti-goat-Cy3 (diluted 1 : 200, Santa Cruz Biotechnology Inc., CA, USA) or Alexa Fluor 488 goat anti-rabbit-Cy2 (diluted 1 : 200, Santa Cruz Biotechnology Inc., CA, USA) secondary antibody for 40 min at room temperature. These sections were mounted with DAPI (Sigma, St. Louis, MO, USA). Images were taken with a laser scanning confocal microscope (Leica TCSSP5; Leica, Wetzlar, Germany).

### 2.3. HEK293 Cell Transfections

Human embryonic kidney (HEK) 293 cells were provided by the cell research center at Basic Medical Peking Union Medical College. HEK293 cells were cultured in a 37°C incubator with 5% CO_2_ and humidified air. The target gene,* HERG* cDNA, and the expression vector, GV314 (GeneChem Co., Shanghai, China), were transfected into cells using Lipofectamine 2000 (Invitrogen, Carlsbad, CA, USA) according to the manufacturer's protocol. Vector GV314 was cotransfected because it contains the marker gene GFP, SV40, CMV promoter, and ampicillin resistance gene. Cells were cultured in Dulbecco's modified Eagle's medium (DMEM; Gibco, MD, USA), 10% fetal bovine serum (Gibco, MD, USA), 1% penicillin-streptomycin (Gibco, MD, USA), and 1% sodium pyruvate (Gibco, MD, USA) for 48–72 h after transfection. Cells were digested with 0.25% trypsin and harvested. Harvested cells were cultured in 35 mm culture dishes.

### 2.4. Patch Clamp Experiments

Cells exhibiting strong fluorescence microscopically (TE2000-U, Niko, Japan) were selected for patch clamp experiments. A patch clamp amplifier (EPC-10, HEKA, Germany) was used to record currents from whole cells. HEK293 cells were bathed in 137 mmol/L of NaCl, 4 mmol/L of KCl, 2 mmol/L of CaCl_2_, 1 mmol/L of MgCl_2_, 10 mmol/L of HEPES, and 10 mmol/L of glucose, pH 7.4. When the patch pipette was filled with the internal pipette solution, the pipette had a resistance of 2.5–4.0 MΩ. The internal pipette solution contained 130 mmol/L of KCl, 5 mmol/L of MgATP, 1 mmol/L of MgCl_2_, 5 mmol/L of EGTA, and 10 mmol/L of HEPES, pH 7.3. The seal resistance was 1-2 GΩ after a successful gigaseal. After membranes were ruptured using negative pressure suction, resistance ranged from 500 to 600 MΩ. Experiments were performed from 25 to 28°C.

Moxifloxacin (Sigma, St. Louis, MO, USA) was dissolved to a concentration of 10 mmol/L with double-distilled water. Further moxifloxacin dilutions were made in the external pipette solution.

### 2.5. Glucose Tolerance Test

This study contained four groups: wild-type mice that received physiological saline, wild-type mice that received moxifloxacin,* HERG* knockout mice that received saline, and* HERG* knockout mice that received moxifloxacin. Each group contained five mice. Moxifloxacin was dissolved in physiological saline. The control groups received physiological saline at the same volume via the same route as the test groups.

Mice were fasted overnight for 16 h but were allowed free access to water. They were given 200 mg/kg of moxifloxacin or the same volume of physiological saline via gavage, and then they were given 2 mg/kg of glucose by intraperitoneal injection. Blood samples were taken from the angular vein at 0, 15, 30, 60, and 120 min after glucose injection. Blood glucose concentrations were assessed using an automatic glucometer (One Touch, LifeScan, USA). Insulin concentrations were measured using a highly sensitive mouse insulin immunoassay kit (Antibody and Immunoassay Services, HKU, China) according to the manufacturer's protocol.

### 2.6. Statistical Analysis

We used a dose-response curve fitted to a Hill equation, *y* = [(*A*_1_ − *A*_2_)/(1 + (*x*/*C*)*n*_*H*_)] + *A*_2_, in which *A*_1_ represents 0 tail current inhibition, *A*_2_ represents 100% inhibition, *C* represents the IC_50_ concentration, and *n*_*H*_ represents the Hill slope. Data analysis was performed using Origin 8.0 and SPSS 19.0 software. All data were presented as mean ± standard error. An independent sample *t*-test was used to compare two sets of data. A one-way ANOVA followed by Tukey's or Dunnett's test was used to compare all groups.  *P* < 0.05  was considered significant.

## 3. Results

### 3.1. Immunohistochemistry of HERG Protein in the Human Pancreas

**Figure 1:**
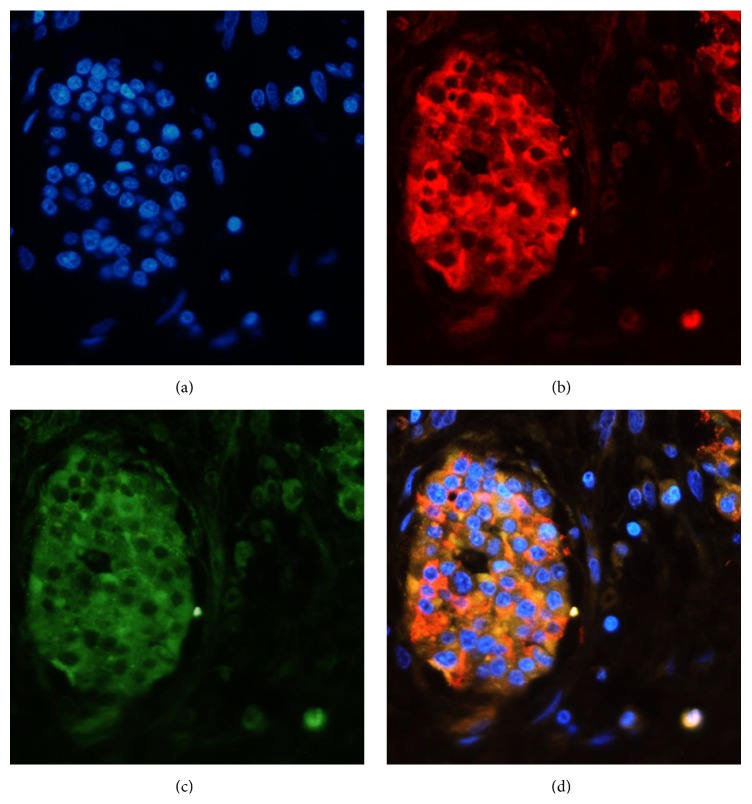
HERG channel proteins expressed in human pancreatic islets. (a) Cell nuclei were stained with DAPI and are illustrated in blue. (b) Insulin immunoreactive cells (red) in the same islet. (c) HERG protein-positive cells (green) in the same islet. (d) Colocalization of insulin and HERG protein.

Pancreatic *β*-cells, which are located in pancreatic islets, were visualized by insulin immunoreactivity (Figure 1(b)). HERG protein-positive cells were located in these islets (Figure 1(c)). Colocalization of insulin and HERG by immunostaining indicated that HERG is expressed in pancreatic *β*-cells (Figure 1(d)).

### 3.2. Moxifloxacin-Induced Inhibition of the HERG Channel in HEK293 Cells

**Figure 2:**
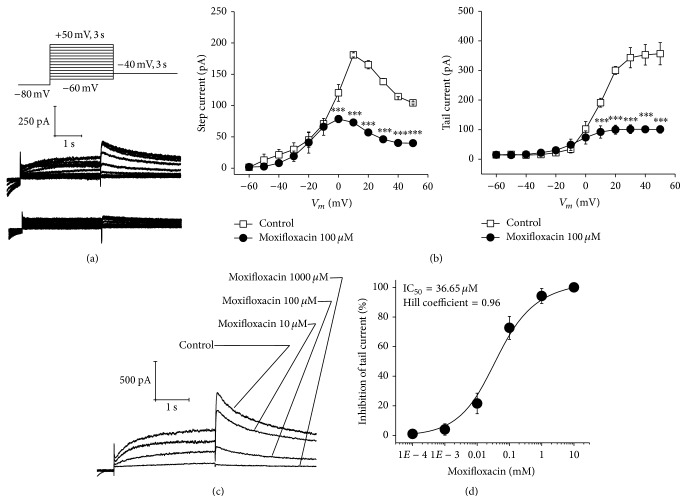
Moxifloxacin-induced inhibition of the HERG channel in HEK293 cells. (a) Cells were voltage-clamped at −80 mV for 1 s, were depolarized from −60 mV to +50 mV for 3 s, and were repolarized to −40 mV for 3 s. HERG currents were recorded in transfected HEK293 cells with and without 100 *μ*mol/L of moxifloxacin. (b) The time-dependent current and peak tail current* I*-*V* curves were recorded before and after cell perfusion with 100 *μ*mol/L of moxifloxacin. (c) Cells were depolarized to a voltage of +20 mV for 3 s using a stepped procedure. Peak tail currents were recorded at different moxifloxacin concentrations (10, 100, and 1000 *μ*mol/L). (d) A moxifloxacin concentration-response curve was fitted to a Hill equation, *y* = [(*A*_1_ − *A*_2_)/(1 + (*x*/*C*)*n*_*H*_)] + *A*_2_, in which *A*_1_ represents 0 tail current inhibition, *A*_2_ represents 100% inhibition, *C* represents the IC_50_ concentration, and *n*_*H*_ represents the Hill slope. ^*∗∗∗*^ *P* < 0.001, *n* = 4.

HEK293 cells were held at −80 mV for 1 s and then were depolarized for 3 s using 10 mV intervals from −60 mV to +50 mV. The current initially increased and then decreased, in a time-dependent manner, as the voltage increased. This reflected the inward rectification of the HERG channel. A tail current was elicited for 3 s at −40 mV and the peak tail current at each voltage represented the degree to which the HERG channel was open at that voltage. After perfusion of the transfected HEK293 cells with 100 *μ*mol/L of moxifloxacin for 5 min, the current was reduced (Figure 2(a)).

Curves* I*-*V* indicated that 100 *μ*mol/L of moxifloxacin significantly reduced the time-dependent and peak tail currents when the voltage was greater than 0 and 10 mV, respectively (Figure 2(b)). Cells were depolarized to +20 mV for 3 s and peak tail currents were recorded at different moxifloxacin concentrations (10, 100, and 1000 *μ*mol/L). Tail currents gradually decreased with the increasing concentrations of moxifloxacin indicating that inhibition by moxifloxacin was concentration-dependent. HERG channel currents were completely inhibited at 1000 *μ*mol/L of moxifloxacin (Figure 2(c)). By plotting moxifloxacin concentration (100 mmol/L) on the  *x*-axis and tail current inhibition (%) on the  *y*-axis, a dose-response curve was generated and the data were fitted to a Hill equation, *y* = [(*A*_1_ − *A*_2_)/(1 + (*x*/*C*)*n*_*H*_)] + *A*_2_. The moxifloxacin IC_50_ was 36.65 *μ*mol/L and the Hill slope, *n*_*H*_, was 0.96 (Figure 2(d)).

### 3.3. Moxifloxacin Decreased the Blood Glucose Concentration and Increased the Serum Insulin Concentration in Wild-Type Mice

**Figure 3:**
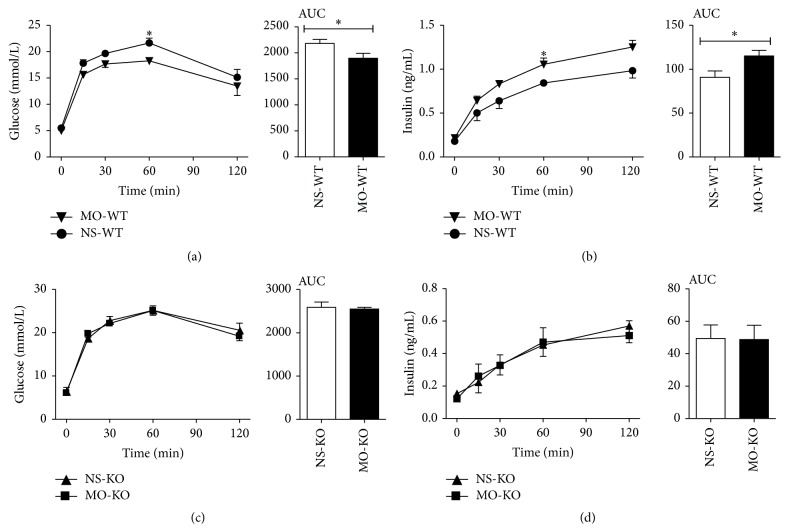
Glucose tolerance test in mice that received moxifloxacin or saline. (a and b) Blood glucose and serum insulin concentrations in wild-type mice that underwent a glucose tolerance test after treatment with 200 mg/kg of moxifloxacin or physiological saline. (c and d) Blood glucose and serum insulin concentrations in* HERG* knockout mice that underwent a glucose tolerance test after treatment with 200 mg/kg of moxifloxacin or physiological saline. ^*∗*^ *P* < 0.05, ^*∗∗*^ *P* < 0.01, *n* = 5.

Blood glucose and insulin concentrations of mice that underwent the glucose tolerance test are shown in Figure 3. In wild-type mice, 200 mg/kg of moxifloxacin led to a reduction in the blood glucose concentration at 60 min (*P* = 0.0346) (Figure 3(a)). Insulin levels in the moxifloxacin group were higher than those in the saline group at 60 min (*P* = 0.0373) (Figure 3(b)). The areas under the blood glucose concentration versus time curve and the insulin concentration versus time curve differed (*P* = 0.0383  and 0.0383, resp.) (Figures 3(a) and 3(b)). There were no significant differences in blood glucose or insulin concentrations in* HERG* knockout mice treated with moxifloxacin or saline (Figures 3(c) and 3(d)).

## 4. Discussion

Increased glucose levels trigger a series of reactions that ultimately lead to insulin secretion by islet *β*-cells. The transformation and utilization of glucose increase the intracellular ratio of ATP/ADP causing K_ATP_ channels to close. The cell membrane is depolarized causing voltage-dependent calcium channels to open, which leads to an increase in the concentration of free intracellular calcium. Then, insulin is released. In addition to K_ATP_ channels [10], other voltage-gated potassium channels are expressed in pancreatic *β*-cells and regulate insulin secretion by repolarizing membranes [11].

Rosati et al. [12] were the first to report that HERG potassium channels are expressed in human islets *β*-cells, and they studied the effect of HERG channels on insulin secretion using electrophysiological and molecular techniques. Yan et al. [9] also found HERG in human islets using reverse transcription-polymerase chain reaction (RT-PCR), but they did not determine its specific distribution. We used immunohistochemistry to show that HERG protein was expressed in human pancreatic *β*-cells.

The HERG inhibitors E-4031, dofetilide, and verapamil impact channel gating to various degrees and can cause currents to be reduced or completely inhibited. Basal insulin secretion in INS-1 *β*-cells was shown to increase upon blockage of ERG channels by E-4031 [13]. We demonstrated that the time-dependent and tail currents for HERG decreased in response to 100 *μ*mol/L of moxifloxacin indicating that moxifloxacin inhibits the HERG channel in HEK293 cells. As moxifloxacin concentrations increased, tail currents were further reduced and completely inhibited at 1000 *μ*mol/L of moxifloxacin. We found that moxifloxacin inhibition was concentration-dependent and that the IC_50_ was 36.65 *μ*mol/L.

Fluoroquinolones can induce life-threatening dysglycemia, but, in the clinical concentration range, fluoroquinolones do not initiate dysglycemia; they rather enhance insulin secretion, which is induced by high glucose concentrations. Fluoroquinolones can block HERG channels and pancreatic *β*-cell K_ATP_ channels; however, the structures of the fluoroquinolones that inhibit HERG channels and K_ATP_ channels differ. The amino group at position C5 of fluoroquinolones plays a major role in blocking HERG channels, whereas the substituents at positions N1, C7, and C8 of fluoroquinolones are responsible for the inhibition of K_ATP_ channels [14]. Inhibition of K_ATP_ channels is required for fluoroquinolone-induced hypoglycemia, but channel inhibition alone does not sufficiently explain this side effect [15]. When HERG channels were selectively blocked by perfusion of islets with antiarrhythmic drugs, insulin secretion increased in the presence of glucose and arginine. A K^+^ current that is pharmacologically and biophysically similar to the HERG current has been reported. These data suggest that HERG channels contribute to the regulation of insulin secretion by human pancreatic *β*-cells. Mutations in HERG channels may explain the hereditary characteristics of hyperinsulinemia of unknown origin [12]. Patients receiving insulin concomitant with moxifloxacin have a significantly higher risk of hypoglycemia in Taiwan [16]. The fluoroquinolone moxifloxacin has been associated with the highest risk of hypoglycemia followed by levofloxacin and ciprofloxacin [16]. Kabbara et al. [17] found that hypoglycemia occurred most often with moxifloxacin compared to levofloxacin and ciprofloxacin.

Ishiwata et al. [18] reported that intravenous injection of 100 mg/kg of moxifloxacin increased the serum glucose concentration and temporarily elevated serum histamine and epinephrine concentrations in fasted rats. Serum glucose, epinephrine, and histamine concentrations remained unchanged when moxifloxacin was administered at 75 mg/kg. Serum insulin concentrations were not altered by moxifloxacin at both 75 and 100 mg/kg. This suggests that moxifloxacin can cause hyperglycemia by inducing histamine and epinephrine release.

In our study, 200 mg/kg of moxifloxacin reduced blood glucose levels in wild-type mice 60 min after the start of the glucose tolerance test; this reduction may be a result of a slight increase in insulin secretion. In contrast, moxifloxacin did not significantly alter blood glucose or insulin levels in* HERG* knockout mice. Serum glucose levels increased and insulin concentrations decreased in* HERG* knockout mice. In a word, this is suggesting that moxifloxacin reduces blood glucose levels and increases insulin secretion via the HERG protein.

The effect of moxifloxacin on HERG channel gating kinetics requires further study. It is also not known whether other channels participate in the moxifloxacin-induced hypoglycemic effect.
